# Upregulation of casein kinase 1ε in dorsal root ganglia and spinal cord after mouse spinal nerve injury contributes to neuropathic pain

**DOI:** 10.1186/1744-8069-5-74

**Published:** 2009-12-18

**Authors:** Eri Sakurai, Takashi Kurihara, Kasumi Kouchi, Hironao Saegusa, Shuqin Zong, Tsutomu Tanabe

**Affiliations:** 1 Department of Pharmacology and Neurobiology, Graduate School of Medicine, Tokyo Medical and Dental University, 1-5-45 Yushima, Bunkyo-ku, Tokyo 113-8519, Japan

## Abstract

**Background:**

Neuropathic pain is a complex chronic pain generated by damage to, or pathological changes in the somatosensory nervous system. Characteristic features of neuropathic pain are allodynia, hyperalgesia and spontaneous pain. Such abnormalities associated with neuropathic pain state remain to be a significant clinical problem. However, the neuronal mechanisms underlying the pathogenesis of neuropathic pain are complex and still poorly understood. Casein kinase 1 is a serine/threonine protein kinase and has been implicated in a wide range of signaling activities such as cell differentiation, proliferation, apoptosis, circadian rhythms and membrane transport. In mammals, the CK1 family consists of seven members (α, β, γ1, γ2, γ3, δ, and ε) with a highly conserved kinase domain and divergent amino- and carboxy-termini.

**Results:**

Preliminary cDNA microarray analysis revealed that the expression of the *casein kinase 1 epsilon *(*CK1ε*) mRNA in the spinal cord of the neuropathic pain-resistant N- type Ca^2+ ^channel deficient (*Ca*_*v*_*2.2*^-/-^) mice was decreased by the spinal nerve injury. The same injury exerted no effects on the expression of *CK1ε *mRNA in the wild-type mice. Western blot analysis of the spinal cord identified the downregulation of CK1ε protein in the injured *Ca*_*v*_*2.2*^-/- ^mice, which is consistent with the data of microarray analysis. However, the expression of CK1ε protein was found to be up-regulated in the spinal cord of injured wild-type mice. Immunocytochemical analysis revealed that the spinal nerve injury changed the expression profiles of CK1ε protein in the dorsal root ganglion (DRG) and the spinal cord neurons. Both the percentage of CK1ε-positive neurons and the expression level of CK1ε protein were increased in DRG and the spinal cord of the neuropathic mice. These changes were reversed in the spinal cord of the injured *Ca*_*v*_*2.2*^-/- ^mice. Furthermore, intrathecal administration of a CK1 inhibitor IC261 produced marked anti-allodynic and anti-hyperalgesic effects on the neuropathic mice. In addition, primary afferent fiber-evoked spinal excitatory responses in the neuropathic mice were reduced by IC261.

**Conclusions:**

These results suggest that CK1ε plays important physiological roles in neuropathic pain signaling. Therefore CK1ε is a useful target for analgesic drug development.

## Background

Neuropathic pain is a complex chronic pain generated by damage to, or pathological changes in the somatosensory nervous system. Neuropathic pain is characterized by the appearance of allodynia (pain perceived in response to normally innocuous stimuli), hyperalgesia (increased responsiveness to painful stimuli) and spontaneous pain [1]. Such abnormalities associated with neuropathic pain state remain to be a significant clinical problem. However, the neuronal mechanisms underlying the pathogenesis of neuropathic pain are complex and still poorly understood [2]. Partly for this reason, attempts to develop new therapeutic agents confront difficulties and the efficacies of currently available drugs for neuropathic pain are reported to be marginal and/or variable for each patient. Thus, development of new strategies leading to pharmacological treatment of neuropathic pain is eagerly awaited. For this purpose, it would be essential to understand the molecular mechanism of the induction and maintenance of neuropathic pain.

In the present study, we have utilized mice lacking N-type voltage-dependent Ca^2+ ^channels (VDCCs) and searched for new neuropathic pain-related molecules. These mice exhibit markedly reduced symptoms of neuropathic pain after spinal nerve injury [3], suggesting a critical role of N-type VDCCs (Ca_*v*_2.2) in the development of neuropathic pain. It is generally believed that changes of gene expression induced by nerve injury contribute substantially to the initiation and maintenance of long lasting neuropathic pain state [4]. Therefore, we have searched for the genes whose expression was altered by spinal nerve injury in the wild-type (*Ca*_*v*_*2.2*^+/+^) and N-type VDCC-deficient (*Ca*_*v*_*2.2*^-/-^) spinal cord using microarray techniques and compared these gene expression profiles. From this preliminary comparative cDNA microarray analysis, we found that the spinal nerve injury down-regulated the expression of *casein kinase 1 epsilon *(*CK1ε*) mRNA in the spinal cord of *Ca*_*v*_*2.2*^-/- ^mice but not of the *Ca*_*v*_*2.2*^+/+ ^mice. CK1 is a serine/threonine protein kinase and has been implicated in a wide range of signaling activities such as cell differentiation, proliferation, apoptosis, circadian rhythms and membrane transport [5-7]. In mammals, the CK1 family consists of seven members (α, β, γ1, γ2, γ3, δ, and ε) with a highly conserved kinase domain and divergent amino- and carboxy-termini. CK1 isoforms were shown to be associated with cytosolic vesicles including small synaptic vesicles and phosphorylated several small synaptic vesicle-associated proteins in neuronal cells [6,8,9]. In the present study, we have tested a possibility that CK1ε plays a role in the maintenance of neuropathic pain state. We first quantified the expression of CK1ε protein and then examined the distribution of this protein in dorsal root ganglia and the spinal cords. Next, we have tested the effects of a CK1 inhibitor on neuropathic pain behaviors. We have also analyzed the effects of the CK1 inhibitor on the excitatory responses in the spinal dorsal horn elicited either by direct activation of postsynaptic glutamate receptors or by presynaptic primary afferent fiber stimulation.

## Results

### Expression of CK1ε in the spinal cord

Using cDNA microarray technique, we previously reported that the expression of ~900 genes out of ~10,000 genes in the *Ca*_*v*_*2.2*^+/+ ^spinal cord was increased more than 1.20-fold by the lumbar L5 and L6 spinal nerve ligation (L5/L6 SNL) injury as compared with sham-operated *Ca*_*v*_*2.2*^+/+ ^mice. Among these genes, we recently suggested that glucocorticoid receptor [10] and peripheral-type benzodiazepine receptor [11] are useful targets in the management of neuropathic pain. On the other hand, we also observed that the expression of ~1,300 genes out of ~10,000 genes was reduced more than 1.20-fold by L5/L6 SNL injury in the *Ca*_*v*_*2.2*^-/- ^spinal cord when compared with sham-operated *Ca*_*v*_*2.2*^-/- ^mice. Because *Ca*_*v*_*2.2*^-/- ^mice showed markedly reduced symptoms of the SNL-induced neuropathic pain [3], we speculated that these down-regulated genes have some contribution to the reduced neuropathic pain symptoms. Among these down-regulated genes, we focused on *CK1ε *in this study, because there has been no information about the expression and function of this molecule in sensory pathway up to this moment, in spite of the fact that CK1 is one of the first serine/threonine protein kinases that were isolated and characterized [5]. The expression of *CK1ε *mRNA was not changed in *Ca*_*v*_*2.2*^+/+ ^mice (1.01-fold) but decreased by 2.81-fold in *Ca*_*v*_*2.2*^-/- ^mice 2 weeks after SNL injury. Quantitative real-time PCR analysis of C57BL/6J mice 2-3 weeks after sham or SNL operation also showed no difference of the *CK1ε *mRNA expression in the spinal cord (n = 4 for sham and SNL, data not shown).

**Figure 1:**
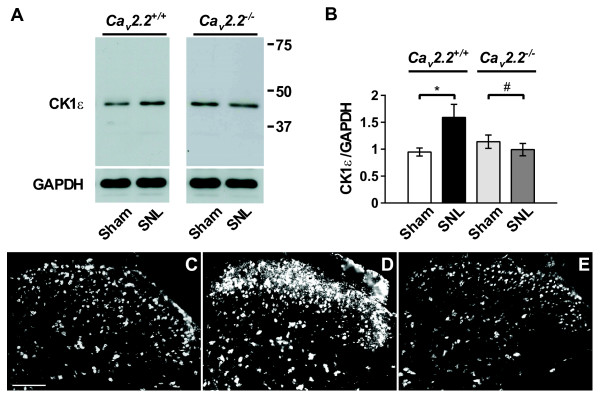
**SNL upregulates CK1ε expression in the spinal cord**. (A) Immunoblot performed with a rabbit polyclonal anti-CK1ε antibody in the *Ca*_*v*_2.2^+/+ ^and *Ca*_*v*_*2.2*^-/- ^spinal cord. L5/L6 segments ipsilateral to the surgery were used. (B) Expression of CK1ε protein, which is normalized by GAPDH loading control. *p < 0.05, SNL *Ca*_*v*_*2.2*^+/+ ^compared with sham *Ca*_*v*_*2.2*^+/+ ^(Student's *t *test), *# p *< 0.05, SNL *Ca*_*v*_*2.2*^-/- ^compared with sham *Ca*_*v*_*2.2*^-/- ^(Student's *t *test); n = 7 in each group. (C-E) Immunofluorescent micrographs of CK1ε-IR in L5 spinal cord from sham- (C) and SNL-operated (D) C57BL/6J mice, and SNL-operated *Ca*_*v*_*2.2*^-/- ^mice (E). Scale bar, 100 μm.

We next examined the expression of CK1ε protein in the spinal cord by immunoblot analyses (Figure 1A and 1B). The CK1ε expression was significantly enhanced in the *Ca*_*v*_*2.2*^+/+ ^spinal cord 2 weeks after SNL injury. In contrast, CK1ε expression in the SNL-operated *Ca*_*v*_*2.2*^-/- ^spinal cord was significantly reduced, which is consistent with the cDNA microarray results.

### Immunohistochemical analysis of CK1ε expression in the spinal cord after spinal nerve injury

We further characterized the distribution of CK1ε-positive cells in the spinal cord of sham and neuropathic mice by immunofluorescence techniques. As shown in Figure 1C-E, the CK1ε protein was found to be broadly expressed in the spinal cord.

Consistent with the immunoblot data, the intensity of CK1ε-immunoreactivity (CK1ε-IR) in the dorsal horn of the spinal cord was increased on the ipsilateral side to the nerve injury.

**Figure 2:**
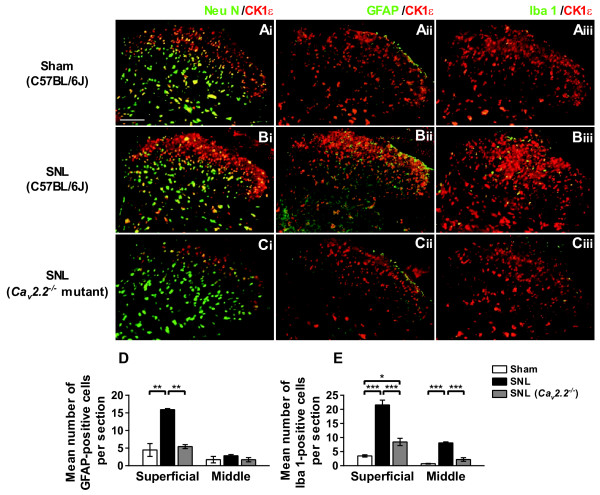
**Immunohistochemical analysis of the L5 spinal cord section**. (A-C) Immunofluorescent micrographs of CK1ε (red) in L5 spinal cord ipsilateral to sham- (A), SNL- (B) operated C57BL/6J mice, and SNL-operated *Ca*_*v*_*2.2*^-/- ^mice (C) co-labeled with NeuN (Ai-Ci), GFAP (Aii-Cii) or Iba 1(Aiii-Ciii) shown in green. Scale bar, 100 μm. (D, E) Mean number of GFAP-positive spinal astrocyte (D) and Iba 1-positive microglia (E). Mouse antibody against CK1ε was used in Aiii-Ciii and rabbit antibody against CK1ε was used in Ai-Ci and Aii-Cii. There was always a tendency that the intensity with the mouse monoclonal antibody is generally stronger and the background is relatively higher than with the rabbit polyclonal antibody, although the specificity of staining was found to be similar between the two antibodies. *p < 0.05, **p < 0.01, ***p < 0.001 (one-way ANOVA followed by Tukey's *post hoc *test); n = 3-4.

To further characterize the localization profile of the CK1ε, we performed double staining of CK1ε with cell-type specific markers. CK1ε-IR was colocalized with a neuronal marker, neuronal specific nuclear protein (NeuN) (Figure 2Ai-Ci), but not with an astorocytic marker, glial fibrillary acid protein (GFAP) (Figure 2Aii-Cii) or a microglial marker, ionized calcium binding adaptor molecule 1 (Iba 1) (Figure 2Aiii-Ciii). Both GFAP and Iba 1 immunostainings were significantly enhanced on the side ipsilateral to SNL injury (Figure 2Bii, Biii, D and 2E), a finding consistent with the literature [12,13]. Intriguingly, SNL-induced enhancements of GFAP- and Iba 1-IR were markedly suppressed in *Ca*_*v*_*2.2*^-/- ^mice (Figure 2Cii, 2Ciii, 2D and 2E). On the other hand, we found SNL injury did not affect the number of NeuN-positive cells in spinal dorsal horn both in wild-type and *Ca*_*v*_*2.2*^-/- ^mice.

**Figure 3:**
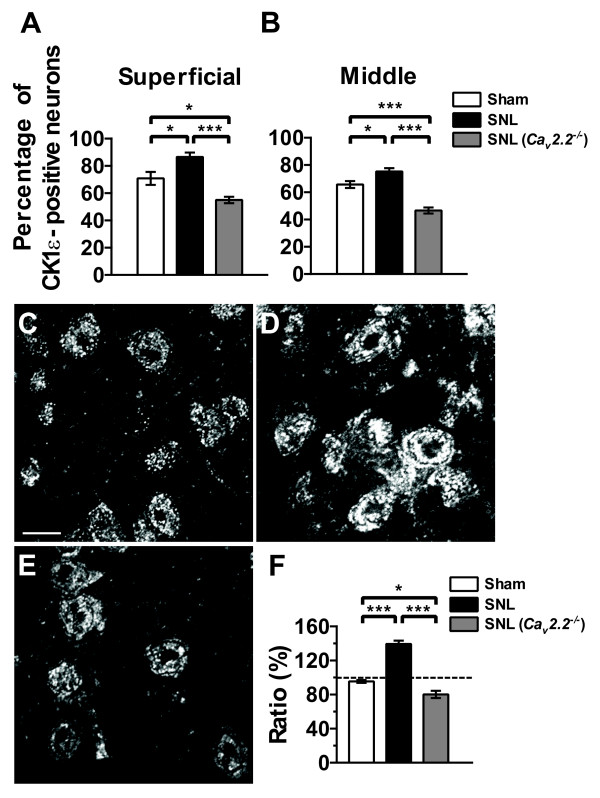
**Effect of SNL injury on the number of CK1ε-positive neurons and CK1ε-IR**. (A, B) Percentage of CK1ε-positive neurons in the superficial (A) and middle (B) layers of the dorsal horn. NeuN-positive cells were regarded as neurons. Each value represents the mean ± SEM of 3-4 determinations. *p < 0.05, ***p < 0.001 (one-way ANOVA followed by Tukey's *post hoc *test). (C-E) Confocal photomicrographs of CK1ε expression in the superficial dorsal horn neurons from sham- (C) and SNL- (D) operated C57BL/6J mice, and SNL-operated *Ca*_*v*_*2.2*^-/- ^mice (E). Scale bar, 10 μm. (F) Averaged intensities of CK1ε-IR in the superficial dorsal horn. *p < 0.05, ***p < 0.001 (one-way ANOVA followed by Tukey's *post hoc *test); n = 3 in each group.

Double staining experiments also revealed that the CK1ε was expressed in 71% (superficial layer) and 66% (middle layer) of the neurons in the L5 dorsal horn of sham-operated spinal cord (Figure 3A and 3B). Two weeks after SNL injury, the percentage was increased to 87% and 75% (superficial and middle layers, respectively) in the L5 dorsal horn ipsilateral to the injury (Figure 3A and 3B) without significant changes in the contralateral side (data not shown). In *Ca*_*v*_*2.2*^-/- ^spinal cord, SNL injury, in contrast, significantly reduced the percentage of the CK1ε-positive neurons in both ipsilateral superficial and middle layers (Figure 3A and 3B). There were no overt differences in the numbers between right and left sides in naive control and between ipsilateral and contralateral sides in sham-operated animals (data not shown).

Although the increase of CK1ε-positive neurons after SNL injury would explain the upregulation of CK1ε protein revealed by the immunoblot experiments, we also tested another possibility that SNL injury increased the expression level of CK1ε protein in the dorsal horn neurons. Confocal microscopic analyses indicated that the expression of CK1ε protein was indeed up-regulated in the ipsilateral superficial dorsal horn neurons of C57BL/6J mice 2 weeks after SNL injury, whereas the same injury slightly reduced the CK1ε expression in *Ca*_*v*_*2.2*^-/- ^spinal cord (Figure 3C-F). CK1ε-IR was detected mostly in the cytoplasm, with only faint staining in the nucleus.

### Immunohistochemical analysis of CK1ε expression in the dorsal root ganglia after spinal nerve injury

To further address the feature of CK1ε expression at neuropathic pain state, we have investigated whether CK1ε is expressed in primary sensory neurons and whether the expression pattern of CK1ε is altered by the SNL injury by immunofluorescence analyses. In agreement with the previous reports [14-16], we observed marked loss (50%) of ipsilateral L5 DRG neurons 2 weeks after the nerve injury when compared with the ipsilateral side of sham operated mice (Table 1). Most dramatic decrease (87%) was observed in large-sized group, and 53% and 38% losses were observed in small- and medium-sized groups, respectively (Table 1). Similar level of cell loss was also observed in SNL-operated *Ca*_*v*_*2.2*^-/- ^mice (Table 1).

**Table 1:** Number of L5 ipsilateral DRG neurons 2 weeks after L5 and L6 spinal nerve injury.

	Small-sized	Medium-sized	Large-sized	Total
Sham C57BL/6J	371.6 ± 31.2	116.2 ± 10.7	18.6 ± 2.90	506.3 ± 42.3

SNL C57BL/6J	176.2 ± 18.4***	72.3 ± 6.64**	2.33 ± 0.59***	250.8 ± 21.9***

SNL *Ca*_*v*_*2.2*^-/- ^mutant	225.3 ± 25.2***	67.7 ± 8.62**	3.17 ± 0.72***	296.1 ± 30.2***

Each value represents the mean ± SEM (n = 12 in each group). **p < 0.01, ***p < 0.001, compared with Sham C57BL/6J (one-way ANOVA followed by Tukey's *post hoc *test). Numbers were obtained using conventional profile counting methods.

**Figure 4:**
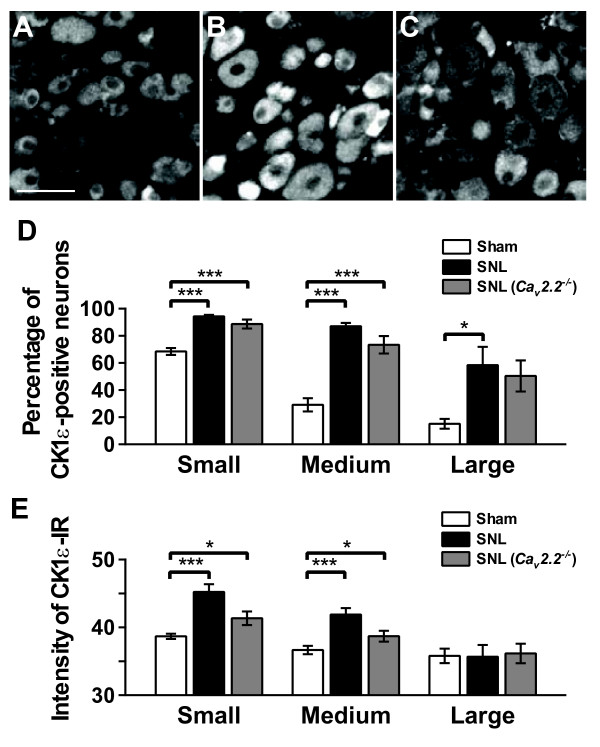
**CK1ε expression in the DRG neurons after spinal nerve injury**. (A-C) Immunofluorescent micrographs showing CK1ε-positive neurons in L5 DRG sections from sham- (A) and SNL- (B) operated C57BL/6J mice, and SNL-operated *Ca*_*v*_*2.2*^-/- ^mice (C). Scale bar, 50 μm. (D, E) Percentages (D) and immunofuorescence intensities (E) of CK1ε-positive neurons in the DRGs. DRG neurons were divided into small-sized (< 600 μm^2^), medium-sized (600-1200 μm^2^), and large-sized (> 1200 μm^2^) groups based on their cross-sectional areas. *p < 0.05, ***p < 0.001 (one-way ANOVA followed by Tukey's *post hoc *test). n = 12.

In L5 DRG, the CK1ε-IR was mainly observed in small- and medium-sized neurons with IR appearing largely in cytoplasm with weak staining in the nucleus (Figure 4). The percentages of CK1ε-positive neurons ipsilateral to sham operation were 68.4%, 29.1% and 15.1% in small-, medium- and large-sized neurons, respectively (Figure 4D). The percentages of CK1ε-positive neurons in the DRG ipsilateral to sham operation were similar to those in contralateral to sham operation and SNL injury, and in naive DRG (data not shown). On the other hand, significant increases in the percentages of CK1ε-positive neurons in the ipsilateral L5 DRG compared with sham group were observed in all size groups 2 weeks after SNL injury (Figure 4B and 4D). Similar changes of CK1ε-positive cell population were also observed in SNL-operated *Ca*_*v*_*2.2*^-/- ^mice (Figure 4C and 4D). Expression level of CK1ε protein was also analyzed by a computerized image analysis system (Figure 4E). When compared with sham group, significant increases in the intensity of CK1ε-IR were observed in the small- and medium-sized neurons of ipsilateral L5 DRG 2 weeks after SNL injury.

**Figure 5:**
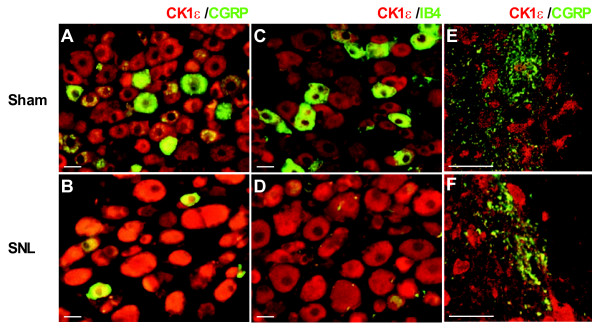
**Expression of CK1ε in subpopulations of the DRG neurons**. (A-D) Immunofluorescent micrographs of L5 DRG sections labelled with CK1ε-IR (red) together with CGRP or IB4 (green) in sham- (A, C) and SNL- (B, D) operated C57BL/6J mice. (E, F) Confocal images of the spinal cord sections labeled with the antibody against CK1ε together with the antibody against CGRP. In the spinal cord, CK1ε (red) is partly colocalized with CGRP (green), a marker for primary afferents, in the superficial dorsal horn in sham- (E) and SNL- (F) operated mice. Scale bars, 20 μm.

Similar extent of increase was also observed in SNL-operated *Ca*_*v*_*2.2*^-/- ^mice (Figure 4E). In contrast, the intensity of CK1ε-IR in large-sized neurons was not significantly changed following SNL injury. To clarify the expression profiles of CK1ε protein in sham and neuropathic mice L5 DRGs, we carried out the double immunofluorescence analysis using CK1ε antibodies together with antibodies against two DRG neuron markers. Small and medium-sized DRG neurons were generally classified into two subgroups, which have been designated as peptidergic and non-pepetidergic [17]. The former expresses two major peptidergic neuromodulators, substance P and calcitonin gene-related peptide (CGRP), and the latter expresses isolectin B4 (IB4)-binding glycoprotein. CK1ε protein was found in both CGRP- and IB4-positive populations (Figure 5). The CK1ε expression was detected in 66.4% of CGRP-positive neurons and 44.9% of CK1ε-positive neurons were CGRP-positive in sham operated DRG neurons (Figure 5). Similarly, the CK1ε expression was detected in 82.3% of IB4-positive neurons and 38.3% of CK1ε-positive neurons were IB4-positive. Similar results were also obtained from naive DRG neurons (data not shown). Two weeks after SNL, a marked decrease of IB4 binding (both the cell number and the intensity) was observed in ipsilateral L5 DRGs (Figure 5D), which is similar to that shown by a previous study [16]. Relatively lesser extent of decrease occurred in the number and the intensity of CGRP-positive small- and medium sized neurons (Figure 5B). In injured DRG neurons, CK1ε-IR was detected in 94% and 91% of CGRP- and IB4-positive neurons, respectively. CGRP-IR was detected in 65% of CK1ε-positive neurons, however, IB4 binding was observed in only 3% of CK1ε-positive neurons. The proportions of CK1ε-expressing cells within CGRP- and IB4-positive neurons in the contralateral DRGs were not different from those of naive and sham-operated DRGs (data not shown). The colocalization of CK1ε with CGRP observed in the superficial dorsal horn area of spinal cord slice preparations (Figure 5E and 5F) suggest that part of the CK1ε protein detected by immunoblot analyses (Figure 1A and 1B) and CK1ε-IR in the spinal cord (Figure 1D) originated from the CK1ε present at the primary afferent fibers and terminals, because CGRP is generally accepted as a pure primary afferent marker [18]. It seems clear that the intensity of CK1ε protein in the primary afferent fibers and terminals is enhanced in CGRP-positive DRG neurons after SNL injury (Figure 5F). Because enhanced protein expression of CK1ε was observed in DRG neurons, we have analyzed mRNA level by quantitative real-time PCR method and found that the 2.03 fold increase of CK1ε mRNA was observed 2 weeks after SNL operation (n = 4 for sham and SNL, *p *< 0.001, data not shown).

### Intrathecal injection of a CK1 inhibitor attenuated neuropathic pain behaviors

**Figure 6:**
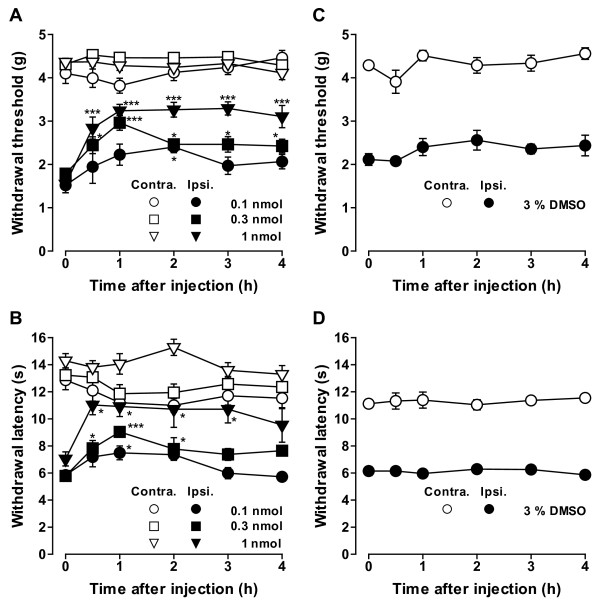
**Effects of CK1 inhibitor IC261 on SNL-induced neuropathic pain behaviors**. (A, B) Effects of intrathecal injection of IC261 on mechanical allodynia (A) and thermal hyperalgesia (B). (C, D) Effects of intrathecal injection of 3% DMSO used for dissolving IC261 on mechanical allodynia (C) and thermal hyperalgesia (D). Paw withdrawal threshold to mechanical stimulation and paw withdrawal latency to thermal stimuli are plotted against the time after intrathecal injection of IC261 or DMSO. Data are mean ± SEM (n = 6-13). *p < 0.05, ***p < 0.001, compared with pre-drug (at 0 h) data (one-way ANOVA followed by Dunnett's *post hoc *test). C57BL/6J mice two weeks after SNL injury were used.

To investigate whether the up-regulation of CK1ε protein is involved in the maintenance of neuropathic pain, we next examined the effects of a CK1 inhibitor IC261 [19]. I.t. injections of IC261 (0.1-1 nmol) dose-dependently increased both withdrawal threshold and withdrawal latency of the hind paw ipsilateral to SNL (Figure 6). Another CK1 inhibitor CKI-7 [20] also showed similar effects (data not shown). These results suggest that blocking the CK1ε activity at the spinal level is effective in reducing mechanical allodynia and thermal hyperalgesia. The maximum effects were observed 0.5-1 h after the injection of IC261 and significant analgesic effects on both mechanical allodynia and thermal hyperalgesia were still observed 3-4 h after the injection of the highest doses used in this study (Figure 6A and 6B). IC261 had no significant effects on the contralateral hind paw (Figure 6A and 6B).

Intrathecal injection of saline or dimethyl sulfoxide (DMSO; 3% in saline) used as a solvent for the drugs did not show any effects on mechanical allodynia and thermal hyperalgesia (Figure 6C and 6D).

### CK1 inhibitor reduced presynaptic primary afferent fiber-evoked spinal excitatory responses

**Figure 7:**
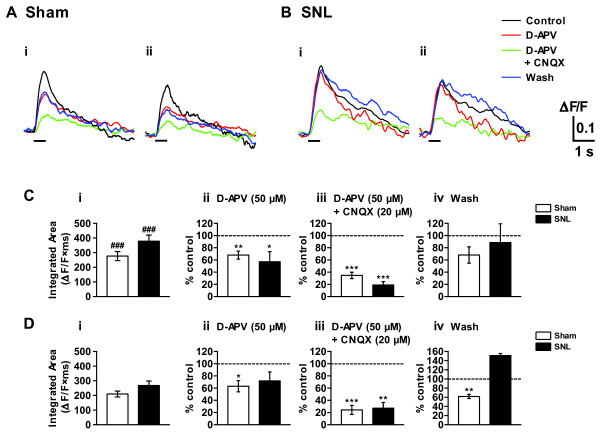
**Primary afferent fiber-evoked spinal excitatory responses in the spinal dorsal horn**. (A, B) Optical responses taken before and after the addition of glutamate receptor antagonists (D-APV 50 μM alone or D-APV 50 μM + CNQX 20 μM) in the perfusion solution for 30 min, and also after a wash of the antagonists. The L5 dorsal root (ipsilateral to sham- or SNL-operation) attached to a transverse slice of sham- (A) or SNL- (B) operated spinal cord was stimulated by a suction electrode. Repetitive stimulation with a high intensity (1 mA, 1 ms current pulses, at 20 Hz for 0.5 s as indicated by the bars) was used to activate both A and C fibers. (C, D) Effects of ionotoropic glutamate receptor antagonists on the dorsal root-evoked excitatory responses recorded in the ipsilateral superficial (Ai, Bi, C) and middle (Aii, Bii, D) layers of dorsal horn. Summary of results, testing the effects of an NMDA-receptor antagonist D-APV (Cii, Dii) and mixture of D-APV and a non-NMDA receptor antagonist CNQX (Ciii, Diii) on the optical response elicited by the dorsal root stimulation. Optical responses were also recorded before drug application (Ci, Di) and after washout of the drugs (Civ, Div). The percentage compared to pre-drug response (as 100%) was shown as % control. ^###^*p *< 0.001, compared with corresponding middle layer response (Student's *t *test). n = 16 and 19 for sham and SNL spinal cord slices, respectively. (Cii-Civ, Dii-Div) *p < 0.05, **p < 0.01, ***p < 0.001, compared with pretreatment control (Student's *t *test). n = 5 for sham and SNL spinal cord slices.

**Figure 8:**
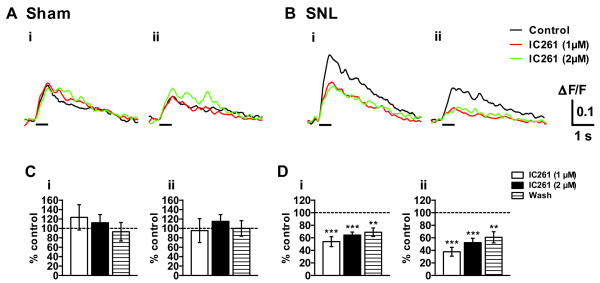
**Effects of IC261 on the primary afferent fiber-evoked spinal excitatory responses**. (A, B) Effects of IC261 on the dorsal root-evoked optical responses were assessed in the sham- (A) and SNL- (B) operated mice. Optical responses were taken before (black lines) and after the addition of IC261 (1 and 2 μM) in the perfusion solution for 30 min (red and green lines, respectively). Ipsilateral superficial (Ai, Bi) and middle dorsal horn (Aii, Bii) were analyzed. (C, D) Summary of results, testing the effects of IC261 on sham- and SNL-operated mice in superficial (Ci, Di) and middle dorsal horn (Cii, Dii). n = 6 and 11 for sham and SNL slices, respectively. The percentage compared to pre-drug response (as 100%) was shown as % control. **p < 0.01, ***p < 0.001, compared with pretreatment control (Student's *t *test).

To explore the mechanism of CK1 inhibitor-induced analgesia at the spinal level, we made L5 dorsal root attached-spinal cord slice preparation from adult mice (9-12 weeks old) and carried out spatio-temporal analyses of the primary afferent fiber-evoked excitatory responses in the dorsal horn by means of imaging techniques using a voltage-sensitive dye 4-(2-(6-(dibutylamino)-2-naphthalenyl)ethenyl)-1-(3-sulfopropyl)-pyridinium hydroxide (di-4-ANEPPS). Repetitive stimulation (10 pulses at 20 Hz) of L5 dorsal root produced prolonged (lasting for 3-4 s) and widely propagating excitatory responses extending from superficial layer to deeper laminae within the ipsilateral L5 spinal dorsal horn (Figure 7). In sham and SNL animals, the magnitude of integrated area of the optical response recorded in the superficial layer of each animal group were significantly larger than those recorded in the corresponding middle layer (Figure 7). Remarkably, the optical responses were not reduced by SNL injury (Figure 7Ci and 7Di), in spite of the fact that 50% of the neurons were lost in DRG. Excitatory synaptic transmission evoked by primary afferents is known to be mainly mediated by glutamate [18,21], and glutamate receptors, especially NMDA receptor, are considered to play important roles in development and maintenance of neuropathic pain [22,23]. Application of an NMDA-receptor antagonist, D-APV (50 μM), suppressed the optical responses in the superficial and middle layers elicited by the nerve stimulation in sham and SNL animals. A perfusion of a solution containing both D-APV (50 μM) and a non-NMDA-receptor antagonist, CNQX (20 μM), further reduced the optical responses in both layers in sham and SNL animals (Figure 7). These results suggest that the activation of glutamate receptors is largely responsible for the induction of the excitatory responses evoked by the repetitive electrical stimulation of the primary afferents. To identify a role of CK1ε in neuropathic pain-related spinal nociceptive transmission, we investigated the effects of the CK1 inhibitor on the dorsal root-evoke optical responses (Figure 8). IC261 (1 and 2 μM) showed significant inhibitory effects on the optical responses in the SNL dorsal horn (Figure 8B and 8D). Similar results were obtained when using another CK1 inhibitor CKI-7 (data not shown).

Interestingly, IC261 did not reduce the dorsal root-evoked optical responses elicited in the dorsal horn of sham mice (Figure 8A and 8C). Vehicle control (0.01 and 0.03% DMSO in ACSF) did not change the optical responses evoked in both superficial and middle layers of naive dorsal horn (data not shown). Thus CK1 inhibitor was found to be effective in reducing spinal excitatory response elicited by the presynaptic electrical stimulation only in neuropathic mice.

### CK1 inhibitor reduced direct NMDA-evoked excitatory responses

To test whether the observed effects of CK1 inhibitor originated from the direct effects on the postsynaptic glutamate receptors, we further examined the effect of IC261 on the optical responses induced by NMDA and glutamate in the SNL spinal cord in the presence of tetrodotoxin (TTX; 0.3 μM).

**Figure 9:**
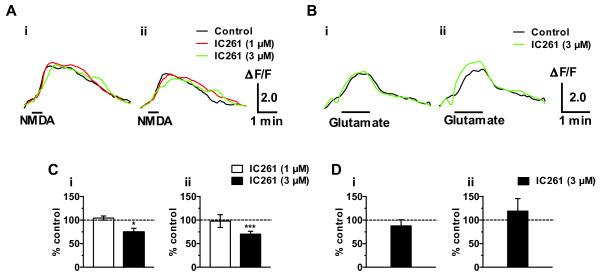
**Effects of IC261 on NMDA- and glutamate- evoked excitatory responses in the spinal dorsal horn**. (A, B) Optical responses taken before (black lines) and after the addition of IC261 (1 and 3 μM, red and green lines, respectively) in the perfusion solution containing TTX (0.3 μM) for 30 min. NMDA- or glutamate-evoked responses were elicited by the perfusion of 300 μM of NMDA for 30 sec (A) or 3 mM glutamate for 1 min (B) (shown as bars). Optical responses were recorded from both superficial (Ai, Bi) and middle (Aii, Bii) layers of dorsal horns ipsilateral to SNL. (C, D) Summary of the results, testing the effects of IC261 (1 μM, n = 5; 3 μM, n = 9) on the NMDA-evoked responses and IC261 (3 μM; n = 4) on the glutamate-evoked responses. The percentage compared to pre-drug response (as 100%) was shown as % control. *p < 0.05, ***p < 0.001, compared with pretreatment control (Student's *t *test).

In both superficial and middle layer of the SNL spinal cord, excitatory optical responses evoked by the bath-application of NMDA (300 μM) for 30 s was not inhibited by 1 μM of IC261 but significantly inhibited by 3 μM of IC261 (Figure 9A). These results suggest that blockade of primary afferent fiber-evoked excitatory responses in the dorsal horn by IC261 (Figure 8B and 8D) was partly caused by the blockade of NMDA evoked responses, because only higher concentration of IC261 was effective. On the other hand, 3 μM of IC261 did not show any effect on the excitatory optical responses evoked by the bath-application of glutamate (3 mM) for 1 min (Figure 9B).

## Discussion

CK1 family constitutes one of the eight major groups of protein kinases in the human and mouse genome [24,25]. However, few physiological roles have been described for CK1 in synaptic transmission. It has been recently reported that metabotoropic glutamate receptors downregulated NMDA receptor-mediated synaptic currents through CK1 dependent activation of protein phosphatases in the striatum [26]. In the present study, we have shown several lines of evidence that CK1ε plays a key role in the maintenance of neuropathic pain induced by spinal nerve injury. Thus, CK1 isoforms expressed in central and peripheral nervous system might display region- and individual cell-specific regulation of synaptic transmission in normal and pathological states.

To our knowledge, this is the first report demonstrating the alteration of the expression pattern of CK1ε in the spinal cord and DRG following spinal nerve injury. After spinal nerve injury, number of CK1ε-positive neurons and the expression level of CK1ε protein were both increased in the superficial and middle layers of ipsilateral L5 dorsal horn. These SNL-induced changes observed in wild-type mice were completely reversed in *Ca*_*v*_*2.2*^-/- ^mice. Furthermore NMDA-evoked excitatory responses and neuropathic pain behaviors were inhibited by IC261. These findings may point the importance of CK1ε-positive neurons within the spinal dorsal horn in neuropathic pain state.

We also found that enhanced GFAP expression possibly reflecting astroglial activation and enhanced Iba 1 expression possibly reflecting microglial migration identified in L5 spinal dorsal horn from neuropathic mice were strongly suppressed in SNL-operated *Ca*_*v*_*2.2*^-/- ^mice. The reason why the glial activation accompanying spinal injury is suppressed in *Ca*_*v*_*2.2*^-/- ^mice is not known at this moment, but this seems to be a reason why *Ca*_*v*_*2.2*^-/- ^mice did not show neuropathic pain symptoms after SNL, because these glial activations have been shown to induce neuropathic pain [12,13]. It would be interesting to explore the mechanism by which N-type Ca^2+ ^channel activation induced by the SNL injury leads to enhanced CK1ε expression in the spinal neuron and glial activation at the spinal dorsal horn in the future study. One plausible mechanism for the enhanced CK1ε expression would be that CK1ε expression may be regulated directly or indirectly by the calcium entry through N-type Ca^2+ ^channel both at the transcriptional and at the translational levels. The calcium dependent transcriptional regulation may include up-regulation of CK1ε mRNA and some miRNAs regulating the translation of CK1ε. If the SNL injury down regulates some of the gene expression including CK1ε besides the activation of N-type Ca^2+ ^channel, mRNA level may not be changed in *Ca*_*v*_*2.2*^+/+ ^mice but will be reduced in *Ca*_*v*_*2.2*^-/- ^mice. Furthermore, if the activation of Ca_*v*_2.2 channel induces translation of CK1ε by the up-regulated regulatory miRNAs or other unknown mechanism, protein level will be increased in *Ca*_*v*_*2.2*^+/+ ^mice but will be decreased in *Ca*_*v*_*2.2*^-/- ^mice. Above presumptive results are exactly what we observed in the actual experiments. However these results are based on the several assumptions that have to be proven experimentally. Furthermore, expression levels of mRNA and protein in DRGs were found to be proportional. Thus further rigorous study would be necessary to clarify the role of N-type Ca^2+ ^channel on the expression of CK1ε.

The effects of nerve injury on the number of DRG neurons have been examined in many different injury models. Although the degree varies in each model presumably due to the differences of the experimental manipulations and the counting methodologies, previous studies in rat [14-16,27] and mouse [28] indicate a loss of neurons in L5 DRG after spinal nerve injury. Our results were found to be consistent with these previous reports. More importantly, we found that both the percentage of CK1ε-positive neurons and the expression level of CK1ε protein were significantly increased in ipsilateral small- and medium-sized L5 DRG neurons after SNL. Furthermore intense expression of CK1ε was found in the primary afferent fibers possibly including presynaptic boutons after SNL injury. These enhanced expression and following activation of CK1ε may be responsible for the apparently normal level of spinal excitatory responses in SNL mice in spite of the fact that more than 50% of the DRG neurons were lost after SNL injury.

Small- and medium-sized DRG neurons are generally considered to correspond to C- and Aδ-fiber neurons, whose axons terminate in the superficial layer of the dorsal horn. On the other hand, large-sized DRG neurons are generally considered to correspond to Aβ-fiber neurons, whose axons terminate in the middle layer of the dorsal horn. Compensation of excitatory responses in the superficial layer of SNL-dorsal horn may be caused by the facilitation of nociceptive transmitter release from the CK1ε-positive C- and Aδ-fiber terminals. In contrast, since the cell loss of large-sized DRG neurons are prominent, the compensation of excitatory responses observed at the middle layer of SNL-dorsal horn may be caused by the indirect effect from the enhanced excitation of superficial layer by the increased input through interneurons linking laminae I-II and laminae III-IV neurons. However, further rigorous study is necessary to verify this hypothesis.

On the other hand, the nature of apparently recovered spinal excitatory responses seems to be very different from those found in normal animals. Firstly, CK1 inhibitor had no effect on the excitatory responses in sham-operated mice but SNL injury turned CK1 inhibitor effective in blocking excitatory responses. Secondly, CK1 inhibitor is effective in blocking neuropathic pain in injured hindpaw without showing any appreciable effect on uninjured hindpaw. Naturally, it is important to identify the target proteins of CK1ε that would induce these changes. Our preliminary experiments trying to identify CK1ε targets resulted in many candidate proteins (data not shown). Further rigorous study is necessary to narrow down and identify the causative proteins.

## Conclusions

The present study demonstrated that SNL injury enhanced the expression of CK1ε protein in spinal dorsal horn and DRG neurons. Furthermore CK1 inhibitor was found to be effective in blocking excitatory synaptic responses in the spinal dorsal horn and neuropathic pain symptoms. These results suggest that CK1ε plays important physiological roles in neuropathic pain signaling, which makes it a useful target for analgesic drug development.

## Methods

### Animals

Male C57BL/6J mice (7-8 weeks old at the time of operation) were purchased from Clea Japan, Inc. (Tokyo, Japan) and housed under controlled temperature (24 ± 1°C) and humidity (55 ± 10%) with a 12-h light-dark cycle with food and water freely available. Experiments were conducted with the approval of the Animal Care Committee of Tokyo Medical and Dental University (approval No. 0090173), and according to the ethical guidelines for the study of experimental pain in conscious animals published by the International Association of the Study of Pain [29].

### Animal model of neuropathic pain

L5/L6 SNL was carried out as described previously [3,10].

### Intrathecal injection

I.t. injection was given in a volume of 5 μl by percutaneous puncture through an intervertebral space at the level of the 5th or 6th lumbar vertebra, according to a previously reported procedure [30,31].

### Behavioral studies

Behavioral studies were conducted in a sound proof room during the light cycle (8:00 a.m.-8:00 p.m.) 2-4 weeks after spinal nerve ligation as described [32]. An investigator, who was unaware of the drug treatment, performed all of the behavioral experiments.

### cDNA microarray analysis

*Ca*_*v*_*2.2*^-/- ^mice were generated and housed as previously reported [3]. Seven *Ca*_*v*_*2.2*^+/+ ^and *Ca*_*v*_*2.2*^-/- ^mice were used for L5/L6 SNL surgery and five *Ca*_*v*_*2.2*^+/+ ^and *Ca*_*v*_*2.2*^-/- ^mice were used for sham surgery. cDNA microarray analysis was performed using the CodeLink™ UniSet Mouse 10K I (GE Healthcare, Piscataway, NJ) following the protocol provided by the manufacturer. Four data sets (*Ca*_*v*_*2.2*^+/+ ^sham, *Ca*_*v*_*2.2*^+/+ ^SNL, *Ca*_*v*_*2.2*^-/- ^sham, *Ca*_*v*_*2.2*^-/- ^SNL) were compared using the CodeLink™ System Software.

### Immunoblot anaysis

Proteins were separated by SDS-PAGE (7.5% gel) and then transferred to a polyvinylidene difluoride membrane (Millipore, Billerica, MA). Anti-CK1ε antibody was used (rabbit; 1: 1000; Santa Cruz Biotechnology, Santa Cruz, CA). We have also tested other anti-CK1ε antibody (mouse; 1: 500; BD Transduction Laboratories, Franklin Lakes, NJ) and found that they showed similar results (single band with same size in immunoblot analysis, data not shown). Immunoreactivity was detected by using the ECL system (GE Healthcare, Buckinghamshire, UK). An anti-glyceraldehyde-3-phosphate dehydrogenase (GAPDH) antibody (mouse, 1:20,000; Chemicon, Temecula, CA) was used to normalize protein loading. Relative intensities of the bands were quantified by using an image analysis system with Image J software, version 1.40 g (National Institutes of Health, Bethesda, MD).

### Immunohistochemistry

Transverse spinal and DRG sections (10 μm) were used. The antibodies used are as follows: CK1ε (rabbit, 1:50; Santa Cruz Biotechnology; mouse, 1:50; BD Transduction Laboratories), CGRP (rabbit, 1:1000; Sigma), NeuN (mouse, 1:100; Chemicon), GFAP (mouse, 1:400; Chemicon) and Iba 1 (rabbit, 1:500, Wako Pure Chemical Industries, Ltd., Osaka, Japan). The sections were then incubated for 2 h at room temperature with Alexa Fluor 488-labeled donkey anti-rabbit IgG (1:1000; Invitrogen, Paisley, UK) or Cy3-conjugated donkey anti-mouse IgG (1:1000; Jackson ImmunoResearch, West Grove, PA). For the detection of IB4 binding, biotinylated IB4 (1:400; Vector Laboratories, Burlingame, CA) and FITC-conjugated extravidin (1:500; Sigma) were used. Sections of a set of control and experimental tissues were concurrently immunostained and images were captured under the same conditions. Control tissue sections, in which the primary antibody was omitted, showed no specific staining. The experiments were carried out at least three times.

Immunofluorescent preparations were examined with a fluorescence microscope (BIOREVO BZ-9000; Keyence Corp, Osaka, Japan).

The numbers of CK1ε-positive neurons were counted in L5 DRGs. On average, five to seven non-adjacent sections of each DRG, where the CK1ε-positive neurons with visible nuclei were equally distributed throughout the rostrocaudal length of the DRG, were randomly selected from 3 to 4 animals in naive control, sham- and SNL-operated groups and numbers of neurons that showed distinctive CK1ε-labeling compared with background labeling were counted as CK1ε-positive by two investigators blinded to the surgical treatment and averaged. The fluorescence intensity was quantified using a 255-level gray scale [33]. To determine the percentage of immunoreactive neurons in each DRG, a threshold of average fluorescence intensity level (for example, 30 in 255-level gray scale for CK1ε) was set by observing several images of normal DRGs. The fluorescence intensity threshold was then applied to all other sections of ipsilateral and contralateral DRGs. The fluorescence intensity and cross-sectional area of CK1ε-positive neurons were quantified using BZ-II analyzer (BZ-H1C software; Keyence Corp, Osaka, Japan). To distinguish cell size-specific changes, we divided the DRG neurons into small-sized (< 600 μm^2^), medium-sized (600-1200 μm^2^), and large-sized (> 1200 μm^2^) groups based on their cross-sectional areas [34,35]. For the co-localization analyses, double stained DRG sections were similarly selected from 3-4 mice, and CK1ε-, CGRP- and IB4-positive cells were counted.

For counting the dorsal horn cells, five to seven sections from the L5 spinal cord segment were randomly selected from each mouse and numbers of distinctive CK1ε-, Neu N-, GFAP- and Iba 1-positive cells in the superficial and middle layers of the dorsal horn were counted by two investigators blinded to the surgical treatment and averaged. The border between superficial and middle layers was delineated according to a representative immunostaining images of protein kinase C type γ (PKCγ) prepared in our laboratory. PKCγ is present in a subpopulation of neurons in the inner part of lamina II and allows a good localization of the border between laminae II and III. The middle layer was defined as dorsal half of deep dorsal horn (laminae III-VI). The number of cells per one section was averaged in each naive control, sham- and SNL-operated animal and the overall means were calculated.

Proportions of CK1ε-positive cells and intensities of CK1ε-IR measured in single- and double-staining were not significantly different in any of the experimental groups. Therefore, data from both single- and double-labeled cells were combined.

### Confocal laser-scanning microscopy

Images of spinal cords stained with the rabbit anti-CK1ε antibody or double labeled with CK1ε (mouse) and CGRP (rabbit) antibodies were collected using a Zeiss LSM 5 Pascal confocal microscope with argon and helium neon lasers (Carl Zeiss Microscopy, Jena, Germany). A × 63, 1.2 NA water-immersion C-apochromatic objective and 2 × zoom value were used for high magnification. The digital images of 20 consecutive z-scan sections (step size approximately 0.5 μm) were analyzed on a computer equipped with an image analysis system (Image J version 1.40 g). CK1ε-IR was quantified using a 255-level gray scale [33]. To quantify CK1ε-IR of the superficial dorsal horn neurons, the immunofluorecence intensities of five randomly selected neurons were quantified using a 255-level gray scale. For each spinal cord section, the ratio of the immunofluorescence intensities of the ipsilateral to the contralateral side was calculated. The ratios for 3-4 non-adjacent sections were averaged in sham- and SNL-operated groups.

### Preparation of spinal cord slices

Spinal cord slices were prepared according to the method described previously [21,36]. Transverse slices (thickness, 600-750 μm) of the L5 spinal segments with the L5 dorsal root attached were prepared and stained with a fluorescent voltage-sensitive dye di-4-ANEPPS (Invitrogen).

### Optical recording

Changes in voltage-sensitive dye fluorescence in the spinal cord were detected using an optical recording system MiCAM02 (Brainvision Inc., Tsukuba, Japan) [37,38]. Repetitive electrical stimulation (current: 1.0 mA, duration: 1 ms) comprised of 10 pulses at 20 Hz were applied through a suction electrode attached to the dorsal root, as described previously [39,40] to induce a long-lasting excitatory component including NMDA receptor transmission. Both A- and C-fibers were thought to be activated by the stimulation mode [41]. For the recording of glutamate receptor agonist-evoked responses in the presence of TTX (0.3 μM), short exposure mode of MiCAM02 recoding and analyzing software (BV analyzer; Brainvision Inc., Tsukuba, Japan) was used to minimize dye bleaching according to the manufacturer's guide. The effect of a pharmacological agent on the nerve- or glutamate receptor agonist-induced responses was evaluated by comparing the averaged magnitude of two or three control responses with the magnitude of response after 25-30 min equilibration for each drug. The concentrations of glutamate receptor antagonists and TTX used were determined according to the previous studies [40,42,43] and our preliminary study.

### Drugs

D-APV, CNQX and IC261 were purchased from Tocris Bioscience, Bristol, UK. Glutamate and NMDA were from Sigma, St. Louis, MO, USA. TTX was from Sankyo Co., Ltd., Tokyo, Japan.

### Statistical analysis

Experimental data are expressed as mean ± SEM. Single comparisons were made using Student's two-tailed paired or unpaired *t*-test. One-way ANOVA followed by the Dunnett's or Tukey's test was used for multiple comparisons. *p *< 0.05 was considered statistically significant.

## Competing interests

Tokyo Medical and Dental University and Japan Science and Technology Agency (JST) hold a shared patent (Japan Patent No. 4227121) based on the results related to but not presented in the paper.

## Authors' contributions

ES carried out all experiments, performed statistical analysis and wrote the manuscript. TK participated in the design of the study, performed optical recording study and wrote the manuscript. KK performed behavioral and immunohistochemical analysis. HS and SZ performed molecular biological study of *Ca*_*v*_*2.2 *mutant mice. TT participated in the design of the study, supervised the experiments and wrote the manuscript. All authors read and approved the final manuscript.
